# Balancing the Cellular Inflammatory-Homeostatic Axis Through Natural Ingredient Supplementation

**DOI:** 10.3390/nu17162587

**Published:** 2025-08-08

**Authors:** Valentina Bordano, Chiara Gerbino, Valentina Boscaro, Patrizia Rubiolo, Arianna Marengo, Stefania Pizzimenti, Marie Angèle Cucci, Stefania Cannito, Jessica Nurcis, Margherita Gallicchio, Simona Federica Spampinato, Luigi Cangemi, Claudia Bocca, Chiara Dianzani, Arianna Carolina Rosa, Elisa Benetti

**Affiliations:** 1 Department of Drug Science and Technology, University of Turin, 10125 Turin, Italy; valentina.bordano@unito.it (V.B.); chiara.gerbino@unito.it (C.G.); valentina.boscaro@unito.it (V.B.); patrizia.rubiolo@unito.it (P.R.); arianna.marengo@unito.it (A.M.); margherita.gallicchio@unito.it (M.G.); simonafederica.spampinato@unito.it (S.F.S.); luigi.cangemi@unito.it (L.C.); chiara.dianzani@unito.it (C.D.); ariannacarolina.rosa@unito.it (A.C.R.); 2 Department of Clinical and Biological Sciences, University of Turin, 10125 Turin, Italy; stefania.pizzimenti@unito.it (S.P.); marieangele.cucci@unito.it (M.A.C.); stefania.cannito@unito.it (S.C.); jessica.nurcis@unito.it (J.N.); claudia.bocca@unito.it (C.B.)

**Keywords:** dietary supplements, inflammation, macrophages, homeostasis, oxidative stress

## Abstract

Background/Objectives: Dietary supplements are sources of nutrients or other substances that added to a healthy lifestyle help to preserve human homeostasis. Since inflammation is one of the major contributors to the alteration of homeostasis, this work investigated the effects of a multi-ingredient dietary supplement on human macrophages, cells involved in the inflammatory response. Methods: THP-1 cells were differentiated into macrophage-like cells and polarized in M_1_ or M_2_ phenotypes. Cell migration was evaluated by Boyden chamber assay; phenotypic markers by qRT-PCR; cytokine release by ELISA and LPS/ATP-induced pyroptosis by LDH assay. The antioxidant properties of the supplement were evaluated in human and mouse fibroblasts by DCF-DA assay. After supplement treatment, cell extracts were analyzed by HPLC-PDA-MS/MS and GC-MS to evaluate the presence of the ingredients. Results: Our results showed that the dietary supplement promoted M_2_ migration and polarization and significantly reduced migration of M_1_. In a model of LPS-induced inflammation in M_0_, it significantly reduced NF-κB activation, COX-2 expression, and cytokine release. The supplement was not a specific inhibitor of NLRP-3, but it was able to modulate LPS priming. In addition, the supplement decreased granulocyte adhesion to HUVEC and reduced the oxidative stress in fibroblasts. The analysis of cell extracts showed the presence of the following ingredients of the formulation inside the cells: CoQ10, spermidine, resveratrol, 5-hydroxytryptophan from *Griffonia simplicifolia* (Vahl ex DC.) Baill., bacosides from *Bacopa monnieri* (L.) Wettst, vit B_2_, B_5_, E acetate. Conclusions: Our results demonstrate how a combination of natural active ingredients may contribute to the maintenance of homeostasis in human cells.

## 1. Introduction

The European Food Safety Authority (EFSA) defined food supplements as concentrated sources of nutrients (i.e., minerals and vitamins) or other substances with a nutritional or physiological effect that are marketed in “dose” form (e.g., pills, tablets, capsules, liquids in measured doses). They may contain a wide range of nutrients, like vitamins, minerals, amino acids, essential fatty acids, fiber, various plants, and herbal extracts [1].

The goal of food supplements is to maintain and support the physiology of the human body. Therefore, they can be intended as substances able to preserve human homeostasis, defined as an individual status in which the values of physiological parameters are in the normal range [2]. They are unable to modify or correct the physiological status: only drugs can restore it.

Currently, food supplements are used by a lot of people worldwide. The growing number of commercially available products as well as the clear desire for people to improve the quality of their life represent the milestones underlying the increasing habit of supplementing the daily diet with efficient and natural solutions [3,4].

Homeostasis represents a fundamental principle in physiology, and significant progress has been made in understanding the molecular mechanisms that govern its regulation. Across all biological systems, it is well established that the values of regulated variables must be constantly monitored and finely adjusted to remain within an optimal range. Accordingly, all homeostatic systems have an essential component named “controller” that has the role of monitoring the value of the regulated variable, comparing it to the reference value and generating a signal for the effector in order to bring the regulated variable closer to the reference value [5]. In our body, typical “controllers” are tissue resident macrophages, mast cells, and somatosensory neurons, all of which monitor various regulated variables of tissue homeostasis. In particular, a crucial role is played by macrophages that are able to act as sentinels by sensing and responding to changing conditions in order to maintain an optimal environment. The role of macrophages in the maintenance of tissue integrity and homoeostasis was already proposed by Metchnikoff, who identified for tissue macrophages an activity focused on preserving homeostasis and tissue integrity. To fulfill this function, macrophages need to suppress inflammatory responses, remove cellular debris from dying and dead cells, and support the restoration of tissue integrity. Macrophages are the only cells ubiquitously present across all organs, positioning them as pivotal regulators of inflammatory processes and as strategic targets for assessing variations in the inflammatory response [6].

Traditionally, homeostasis and inflammation are viewed as opposing states in biological systems, with homeostasis reflecting physiological stability, while inflammation is typically associated with diseases or imbalances. However, a controlled inflammatory response is beneficial (for example, in providing protection against infections), but it can become detrimental if dysregulated, becoming pathological and sometimes chronic. The disruption of cellular and tissue homeostasis is a common condition associated with inflammation [7,8,9].

On this basis, dietary supplements could exert beneficial effects by counteracting the establishment or the exacerbation of inflammatory responses, thus preserving human homeostasis.

Accordingly, our research aimed to evaluate whether a multi-component dietary supplement could exert positive effects on human cells engaged in the regulation of inflammation and the preservation of homeostatic balance.

## 2. Materials and Methods

### 2.1. Formulation and Preparation of the Multi-Ingredient Supplementation Used in This Study

- The dietary supplement object of this study is composed by coenzyme Q10 (CoQ10), spermidine, D-ribose, resveratrol, phosphatidylserine, griffonia dry extract [*Griffonia simplicifolia* (Vahl ex DC) Baill.] seed, bacopa dry extract [*Bacopa monnieri* (L.) Wettst.] grass, vitamins B_1_, B_2_, B_3_, B_5_, B_6_, B_9_, B_12_, C, E, selenium, and zinc. In Table S1 there is the quantity for each ingredient relative to the recommended dose.
- For cell experiments we dissolved the daily recommended dose in the minimum necessary volume (30 mL) of distilled water for the hydrophilic component and of DMSO for the lipophilic one. Before each experiment, the dietary supplement was prepared by adding two parts of the hydrophilic components to one part of the lipophilic one (to reproduce the correct proportions present in the capsules) and then we performed the dilutions.

### 2.2. Cells Culture

THP-1 monocytes were maintained in RPMI 1640 medium (Aurogene Srl, Rome, Italy), supplemented with 2 mM L-glutamine, 100 μg/mL penicillin–streptomycin, and 10% (*v*/*v*) fetal bovine serum (FBS; all from Aurogene Srl), at 37 °C in a humidified incubator with 5% CO_2_. Differentiation into macrophages was induced by exposure to 50 ng/mL phorbol 12-myristate 13-acetate (PMA; Sigma-Aldrich, St. Louis, MO, USA) for 48 h. Subsequently, polarization into either pro-inflammatory (M_1_) or anti-inflammatory (M_2_) phenotypes was achieved by 24 h treatment with 100 ng/mL lipopolysaccharide (LPS; Sigma-Aldrich) plus 20 ng/mL interferon-γ (IFN-γ; R&D Systems, Minneapolis, MN, USA), or 20 ng/mL interleukin-4 (IL-4; Thermo Fisher Scientific, Waltham, MA, USA) plus 20 ng/mL interleukin-13 (IL-13; Thermo Fisher Scientific), respectively [10,11].

Primary human umbilical vein endothelial cells (HUVECs) were obtained through 1% trypsin digestion of umbilical veins and cultured in M199 medium (Euroclone S.p.A., Milan, Italy), supplemented with 20% fetal calf serum (FCS), 100 U/mL penicillin, 100 μg/mL streptomycin, 5 IU/mL heparin (Techdow, Milan, Italy), 12 μg/mL homemade bovine brain extract, and 200 mM glutamine. Cells were expanded to confluence in culture flasks and used between passages 2 and 5. Experimental procedures involving HUVECs were approved by the Ethics Committee of “Presidio Ospedaliero Martini” in Turin (protocols 263-07/NF and pr_SanPlac/16) and were conducted in full compliance with the Declaration of Helsinki.

Polymorphonuclear neutrophils (PMNs) were isolated from peripheral venous blood collected from healthy volunteers, following written informed consent and in compliance with the ethical principles outlined in the Declaration of Helsinki. PMNs were separated by standard dextran sedimentation followed by Histopaques 1077 (Sigma-Aldrich) gradient centrifugation. Residual erythrocytes were eliminated via hypotonic lysis, and PMNs were subsequently resuspended in M199 medium. The resulting cell suspension consistently exhibited purity and viability exceeding 95%, as verified by the trypan blue exclusion assay.

Human Dermal Fibroblasts (HDFs) and murine L929 fibroblast cells were maintained in Dulbecco’s Modified Eagle Medium (DMEM; Euroclone S.p.A.), enriched with 2 mM L-glutamine, 100 μg/mL penicillin–streptomycin, and 10% (*v*/*v*) fetal bovine serum (FBS; all from Aurogene Srl). Cultures were incubated at 37 °C in a humidified atmosphere containing 5% CO_2_.

### 2.3. Measurement of Cell Viability

THP-1 or HUVEC were plated (2 × 10^4^ cells/well and 5 × 10^3^ cells/well, respectively) in 96-well culture plates. THP-1 cells were exposed to PMA for 48 h. Subsequently both cells were treated with serial dilutions of the supplement (1:1000, 1:750, 1:500, 1:250, 1:100) for 24 or 48 h. Cell growth in sub-confluent cultures was assessed using the colorimetric MTT assay (3-(4,5-dimethylthiazol-2-yl)-2,5-diphenyltetrazolium bromide; Sigma-Aldrich), following established protocol [12]. Results were confirmed by determining cell density, as previously described [10,13].

### 2.4. Cell Motility Assay (Boyden Chamber Assay)

To evaluate cell migration via the Boyden chamber assay (BD Biosciences, San Jose, CA, USA), THP-1 cells (5 × 10^3^ cells/well) previously differentiated and polarized into M_1_ and M_2_ macrophages were incubated with or without the supplement at various dilutions for 24 h. Subsequently, cells were seeded on the apical surface of Matrigel-coated filters (50 μg/mL; 8.2 mm diameter, 5 μm pore size; Neuro Probe Inc., BIOMAP, Gaithersburg, MD, USA) in a serum-free medium. The basolateral compartment was filled with a medium containing 30 nM C–C motif chemokine ligand 7 (CCL7; ImmunoTools GmbH, Friesoythe, Germany), serving as a chemoattractant. After a 6 h incubation, non-migrated cells on the apical side were gently removed with cotton swabs, and migrated cells on the lower filter surface were fixed and stained using methanol-crystal violet. Migratory cells were counted across all microscopic fields using an inverted microscope (n = 7).

### 2.5. Western Blot Analyses

Western blot analyses were performed by loading approximately 15 μg of total protein per lane, following the protocol previously reported [13,14]. After membrane blocking, PVDF filters were incubated overnight at 4 °C with a primary antibody targeting p65 NF-κB (dilution 1:1000, Cell Signaling Technology, Danvers, MA, USA) or with an antibody against COX-2 (dilution 1:1500, Bioss Antibodies, Woburn, MA, USA). To verify consistent protein loading across samples, membranes were probed with a monoclonal antibody against GAPDH (dilution 1:2000, Cohesion Biosciences, London, UK) for cytosolic extracts or with anti-Laminin beta 1 (dilution 1:500, CliniSciences, Rome, Italy) polyclonal antibody for nuclear extracts. The detection of target proteins was achieved using a horseradish peroxidase—conjugated secondary antibody (1:2000 dilution, Cell Signaling Technology), applied for 1 h at room temperature.

### 2.6. RNA Isolation and Quantitative Real-Time PCR (RT-qPCR)

Total RNA was isolated using TRI Reagent^®^ (Sigma-Aldrich) in accordance with the manufacturer’s protocol. Synthesis of complementary DNA and subsequent quantitative real-time PCR (qRT-PCR) were performed on cell-derived samples, as previously described [15]. Gene expression levels were quantified using the SYBR^®^ Green detection method. Amplification of the housekeeping gene GAPDH (glyceraldehyde-3-phosphate dehydrogenase) was carried out in parallel across all PCR assays. Primer sequences used for qRT-PCR are listed in the Table 1 below:

mRNA amounts were calculated according to the threshold cycle of individual genes and their relative expression was quantified by serial dilutions of the amplified products compared with external standard curves of the reference genes containing known amounts of each gene product. The results were expressed as a relative ratio of the target to the housekeeping gene using the Light Cycler Relative Quantification software 4.05 (Roche Diagnostics, Monza, Italy). Each sample was analyzed in triplicate to determine mRNA expression levels. The specificity of PCR amplification was assessed via melting curve analysis and further validated by agarose gel electrophoresis followed by ethidium bromide staining.

### 2.7. Measurement of Cytokines Release

Macrophage-like cells, obtained from PMA-differentiated THP-1 cells, were pre-treated for 24 h with the supplement (1:2000, 1:1000, 1:500) and then with LPS (100 ng/mL; 24 h). At the end of incubation, cell culture supernatants were collected and the levels of IL-1β and TNF-*α* were quantified with an enzyme-linked immunosorbent assay (ELISA) kit (4A Biotech Co., Beijing, China) according to the manufacturer’s instructions.

### 2.8. Pyroptosis Assay

THP-1 cells were plated (9 × 10^4^ cells/well) in 48-well culture plates and differentiated by treatment with PMA (50 nM, 24 h). M_0_ cells were washed twice with phosphate-buffered saline (PBS) and primed with LPS (10 μg/mL, 4 h) in a serum-free medium. Pyroptosis was induced by NLRP-3 activation treating cells with ATP (5 mM, 90 min; Sigma-Aldrich, St. Louis, MO, USA). Cells were treated with serial dilutions of the supplement (1:2000, 1:1000, 1:500) either before LPS priming or 1 h before ATP treatment. LDH release was quantified by using the CytoTox 96 nonradioactive cytotoxicity assay (Promega Corporation, Madison, MI, USA) and the absorbance was measured using Victor X4 (PerkinElmer, Waltham, MA, USA) at λ = 490 nm. LDH release was expressed according to the manufacture’s instruction.

### 2.9. Adhesion Assay

HUVEC grew to confluence in 24-well culture plates and then treated with serial dilutions of the supplement. After 48 h, HUVEC were stimulated with TNF-α (10 nM) for a further 24 h, in order to activate the expression of adhesion molecules, and then the adhesion of PMNs was evaluated. PMNs were labeled with fluorescein diacetate (5 µg/mL, Sigma-Aldrich) for 30 min at 37 °C, washed with Buffered Saline Solution (BSS), and plated at 7 × 10^4^ cells/well on HUVEC for 45 min. After incubation, non-adherent PMNs were removed by washing with 1 mL of M199. Fluorescence-based image analysis was performed at the center of each well [16]. Quantification of adherent cells was carried out using Image Pro Plus micro-imaging software (Media Cybernetics, Rockville, MD, USA, version 5.0). Each experimental condition was tested in quadruplicate, yielding a standard error below 10% across all replicates. Data are presented as a percentage of adhesion inhibition versus the value achieved with the positive control (TNF-α treatment, 100%). Baseline adhesion values were obtained from untreated HUVEC samples.

### 2.10. Measurement of Cellular Oxidative Stress

Fibroblasts were seeded at a density of 2 × 10^5^ cells per well in 6-well plates containing 2 mL of medium supplemented with serum. Intracellular oxidative stress was assessed by incubating cells with dichlorodihydrofluorescein diacetate (DCF-DA; Invitrogen, Carlsbad, CA, USA). Fluorescence signals corresponding to 2,7-dichlorodihydrofluorescein (DCF) and 2-hydroxyethidium were quantified using an Accuri C6 FACScan flow cytometer (Becton Dickinson Italia, Milan, Italy).

### 2.11. Chemical Analysis

The dietary supplement and the samples obtained from the cell-based experiments were subjected to chemical characterization in order to identify the main components.

Sample preparation and chemical analysis were adapted based on the different physicochemical properties of the various compounds. The components monitored in the following experiments, listed in Table S1, were selected based on their detection limit, which was determined by analyzing the dietary supplement at various dilutions.

#### 2.11.1. Chemicals

Methanol MS grade, N,O-bis (trimetilsilyl) trifluoroacetamide (BSTFA), methoxyamine hydrochloride (MOX), pyridine, formic acid and bacoside A (mixture of bacoside A3, bacopaside II, bacopaside X, bacopasaponin C) were from Merck (Darmstadt, Germany). Deionized water (18.2 MW cm) was supplied by a Milli-Q purification system (Millipore, Burlington, MA, USA). The pure standards used for compound identification were by Acfp. All solvents used are of analytical purity.

#### 2.11.2. Sample Preparation

For the cell-based experiments, cell extracts untreated and treated with dietary supplement (1:100, 24 h) were freeze-dried and subjected to specific sample preparation based on the compounds to be monitored. The dietary supplement was analyzed likewise as a reference.

Detailed information is provided in Table 2. For the high-performance liquid chromatography coupled with diode array and mass spectrometry detectors (HPLC-PDA-MS/MS) analyses, different dilution solvents were employed, and the samples were filtered with a 0.20 μm hydrophobic polytetrafluoroethylene (PTFE) or hydrophilic polyvinylidene fluoride (PVDF) filter (CPS Analitica, Milano, Italy). For the analysis of ribose, the samples were subjected to a derivatization step prior gas chromatography coupled with mass spectrometry (GC-MS) analysis. Eighty μL of MOX in pyridine (15 mg/mL) was added to a weighted amount of the sample and the solution was heated to 60 °C for 30 min. Then 120 μL of BSTFA was added and the solution was heated again to 60 °C for 30 min to obtain the trimethylsilyl derivative.

#### 2.11.3. HPLC-PDA-MS/MS Analysis

Analyses were carried out using a Shimadzu Nexera×2 liquid chromatography system coupled to an SPD-M20A photodiode array detector (Shimadzu, Tokyo, Japan), in tandem with a Shimadzu LCMS-8040 triple quadrupole mass spectrometer equipped with an electrospray ionization (ESI) source (Shimadzu, Tokyo, Japan).

As reported in Table 2 different stationary phases and gradient programs were used for the analysis of the components present in the dietary supplement.

Water/formic acid (999:1, *v*/*v*) was used as mobile phase A and methanol/formic acid (999:1, *v*/*v*) as mobile phase B for all analyses. The flow rate was set to 0.3 mL/min and the column was kept at 30 °C. The UV spectra were acquired over the wavelength range of 220–450 nm.

The MS parameters were as follows: the temperature of the heating block was 200 °C; the temperature of the desolvation line (DL) was set at 250 °C; the flow rate of nebulizer gas and drying gas was 3 L/min and 15 L/min, respectively. Mass spectra were acquired in positive and negative full scan mode over the range of 100–1000 *m*/*z*, event time 0.5 s.

The main components were identified by comparing their retention times, UV and MS spectra with those of pure reference compounds.

The presence of the components in the cell extract treated with the supplement was determined by comparing the peak areas of the selected compounds (in UV or MS in SRM and SIM mode) in the treated sample with the untreated sample (which served as a control). For resveratrol and CoQ10, the wavelength was chosen based on the maximum of absorption for each compound. For the bacosides, the ions monitored in Single Ion Monitoring (SIM) were selected based on the characteristic deprotonated molecules, considering their adduct products with formic acid in negative ionization mode, i.e., *m*/*z* 943 and 973. For the other analytes, the transitions monitored in Selected Reaction Monitoring (SRM) in positive ionization mode (collision energy: −35.0 V, dwell time: 5) were chosen based on the fragmentation profile of the compounds analyzed in Product Ion Scan mode (collision energy: −35.0 V, event time: 0.2 s).

#### 2.11.4. GC-MS Analysis

The samples were analyzed by GC-MS (Agilent 6890 GC unit coupled to an Agilent 5973 MSD, Agilent, Little Falls, DE, USA), for the detection of Ribose (after derivatization) and vitamin E acetate. The separation was performed on a column 5% Phenyl polydimethylsiloxane (30 m, dc 0.25 mm, df 0.25 μm). Helium was used as the carrier gas at a constant flow rate of 1 mL/min. The injector and mass spectrometer detector temperatures were set at 250 °C and 280 °C, respectively. Electron impact ionization was performed at 70 eV, with a scanned mass range between 50 and 800 *m*/*z*. The oven temperature program included an initial hold at 50 °C for 1 min, a ramp to 300 °C at 3 °C/min, followed by a 10 min hold at 300 °C. Compound identification was achieved by matching the obtained mass spectra with reference spectra from the Wiley and NIST databases, and further confirmed through co-injection of authentic standards.

The presence of the components in the cells was monitored by comparing the peak areas of the compounds in SIM (*m*/*z* 307 for ribose and *m*/*z* 165 for vitamin E acetate) of the treated and untreated samples. Data was processed using Agilent MSD ChemStation (Agilent Technologies).

### 2.12. Data Analysis

Data are expressed as mean ± standard error of the mean (SEM). Statistical comparisons were performed using Student’s *t*-test when appropriate or one-way ANOVA followed by Bonferroni post hoc correction. Analyses were conducted using GraphPad Prism software, version 10.0 for Windows (GraphPad Software, San Diego, CA, USA). Differences were considered statistically significant at *p* < 0.05. Statistical significance was denoted as follows: * *p* < 0.05, ** *p* < 0.01, *** *p* < 0.001. When relevant, additional symbols were used to indicate comparisons between treatment groups and the positive control, in cases where asterisks already denote comparisons between negative and positive controls. Specifically, # indicates comparison vs. LPS-treated group, and ° indicates comparison vs. H_2_O_2_-treated group.

## 3. Results

### 3.1. Effect of the Dietary Supplement on Cell Growth in Macrophage-like Cells

**Figure 1:**
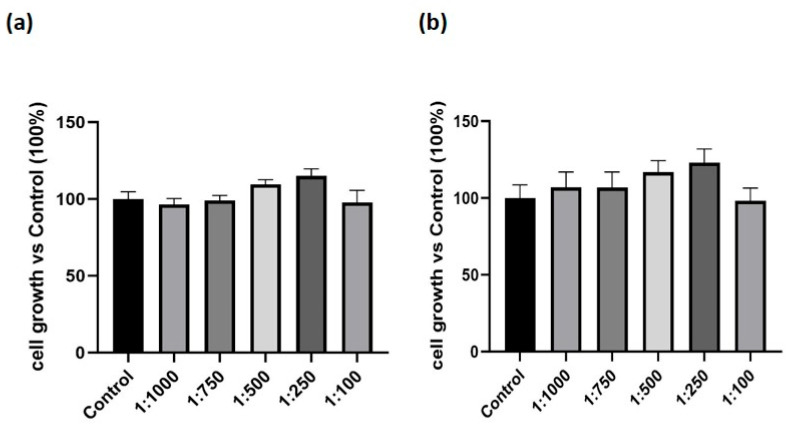
Effect of the dietary supplement on cell growth in macrophage-like cells. M_0_ macrophages were exposed to either vehicle alone (Control) or to serial dilutions of the supplement. (**a**) cell growth at 24 h, measured using the MTT assay. (**b**) cell growth at 48 h, measured using the MTT assay. Results are expressed as percentage relative to Control (set at 100%). Data are expressed as mean ± SEM of three independent experiments conducted in triplicate.

To assess the potential cytotoxic effects of the supplement, THP-1 cells differentiated into M_0_ macrophages were exposed to either vehicle alone or serial dilutions of the supplement (1:1000, 1:750, 1:500, 1:250, 1:100) for 24 h or 48 h. Cell growth was evaluated by MTT assay. As shown in Figure 1, no changes in cell growth were observed at either point when compared to control cells. These findings indicate that the supplement did not impair the growth rate of our cells.

### 3.2. Effect of the Dietary Supplement on Macrophage Migration and Polarization

**Figure 2:**
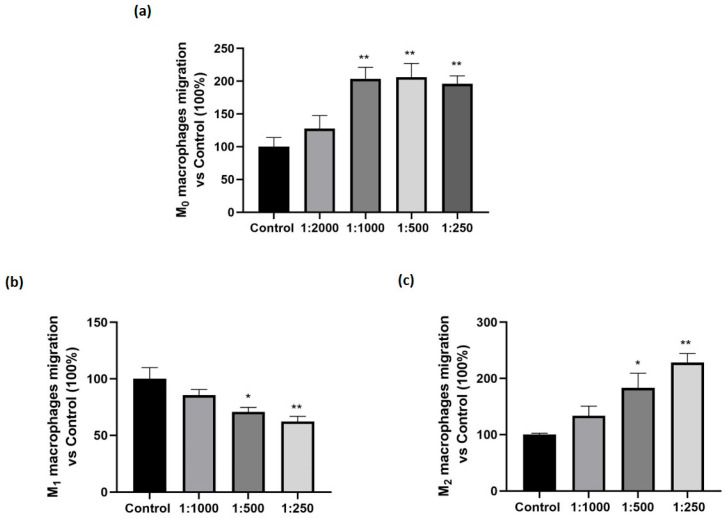
Effect of the dietary supplement on macrophage-like cells migration. Macrophages differentiated into M_0_, M_1_, M_2_ phenotypes were exposed for 24 h to either vehicle alone (Control) or serial dilutions of the dietary supplement, in the presence of the chemotactic stimulus CCL7 (30 nM). (**a**) migration of M_0_ macrophages. (**b**) migration of M_1_ macrophages. (**c**) migration of M_2_ macrophages. Cell migration was assessed using the Boyden chamber assay. Results are expressed as percentage relative to Control (set at 100%). Data are expressed as mean ± SEM of three independent experiments conducted in triplicate. * *p* < 0.05, ** *p* < 0.01 vs. Control.

The potential ability of the supplement to modulate the migration of M_0_, M_1_, and M_2_ macrophage-like cells, was evaluated by the Boyden chamber assay. Cells were pre-treated with the supplement for 24 h. As a chemotactic factor, we used CCL7 (30 nM, 6 h), a chemokine suitable for inducing chemotaxis of all macrophage phenotypes [17]. As shown in Figure 2, the treatment significantly enhanced the migration of M_0_ macrophages starting from the dilution of 1:1000 (panel a). Interestingly, the supplement exerted differential effects depending on the macrophage phenotype: it reduced the migration of M_1_ macrophages (the pro-inflammatory one, panel b) and increased the migration of M_2_ macrophages (the anti-inflammatory phenotype, panel c). For both the phenotypes the effect was significant starting from the dilution of 1:500.

**Figure 3:**
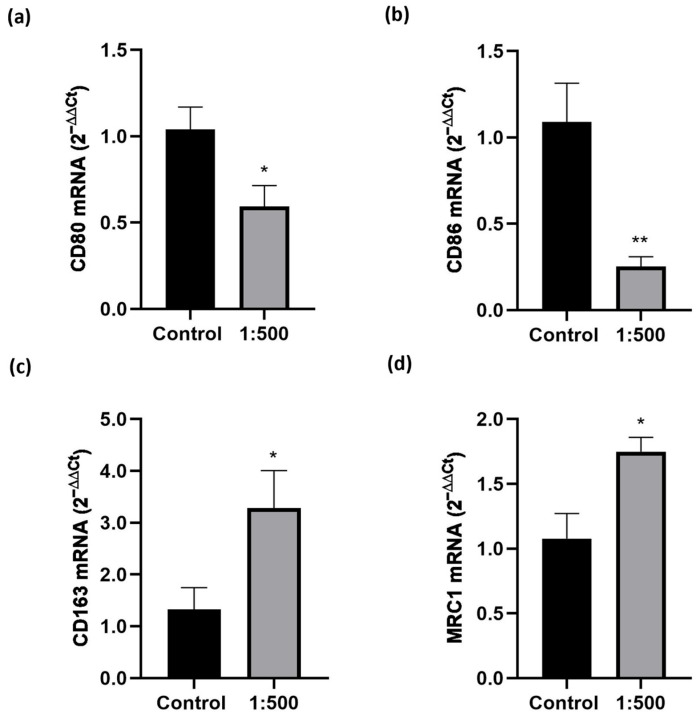
Effect of the dietary supplement on M_1_ and M_2_ phenotypic markers. THP-1 cells were differentiated in M_0_ macrophage-like cells and treated with the supplement (1:500 dilution) for 24 h. Gene expression levels of selected phenotypical markers were assessed by Real-Time PCR. (**a**) CD80 expression, (**b**) CD86, (**c**) CD163, and (**d**) MRC1. Gene expression values were normalized to the housekeeping gene GAPDH and presented relative to untreated control cells (Control). Data are expressed as mean ± SEM of three independent experiments run in triplicate. * *p* < 0.05, ** *p* < 0.01 vs. Control.

The unexpected, marked increase in M_0_ macrophage migration prompted us to investigate the effects of the dietary supplement on M_0_ polarization. The results (Figure 3) were very clear, showing that treatment with the dietary supplement significantly enhanced the expression of M_2_ phenotypic markers, such as MRC1 and CD163, while significantly reducing the expression of M_1_ pro-inflammatory markers, like CD80 and CD86.

### 3.3. Effect of the Dietary Supplement on LPS-Induced Inflammation

**Figure 4:**
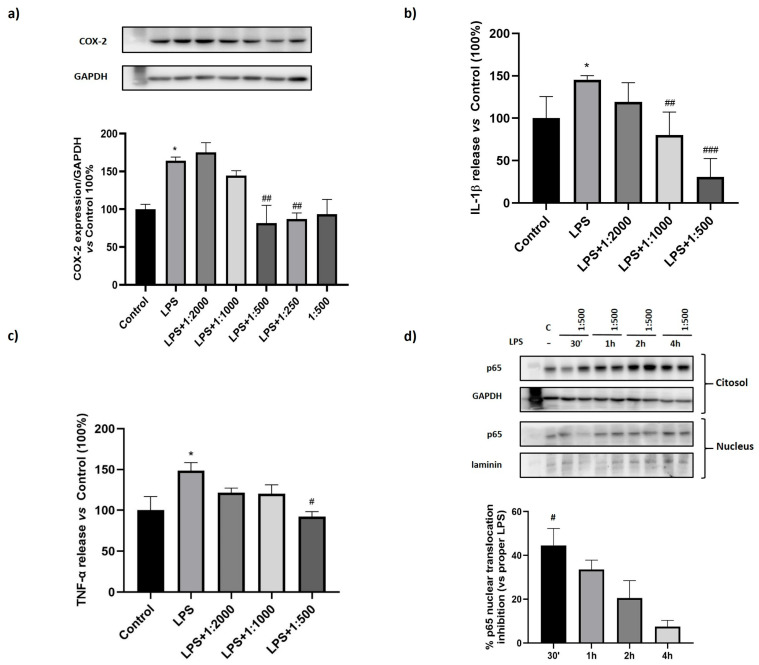
Effect of the dietary supplement on LPS-induced inflammation. THP-1 cells were differentiated in M_0_ macrophage-like cells, pre-treated with serial dilutions of the dietary supplement for 24 h and then with LPS (100 ng/mL) for 24 h (**a**–**c**) or for 30 min–4 h (**d**). (**a**) COX-2 expression evaluated by Western-blot; (**b**) IL-1β levels measured in the conditioned media by ELISA; (**c**) TNF-α levels measured in the conditioned media by ELISA; (**d**) nuclear translocation of p65 NF-κB evaluated by Western blot. Data are expressed as mean ± SEM of three independent experiments run in triplicate. * *p* < 0.05 vs. Control; ^#^ *p* < 0.05, ^##^ *p* < 0.01 and ^###^ *p* < 0.001 vs. LPS.

To investigate the potential anti-inflammatory properties of the dietary supplement, we employed an acute LPS-induced inflammation model. Macrophage-like cells were treated for 24 h with serial dilutions of the supplement (1:2000, 1:1000, 1:500, 1:250), followed by stimulation with LPS (100 ng/mL) for an additional 30 min–4 h to evaluate NF-κB activation or for 24 h to assess COX-2 expression or cytokine release. Cell extracts were then analyzed by Western blot and conditioned media by ELISA. The results showed that, as expected, LPS induced NF-κB activation; however, treatment with the dietary supplement significantly inhibited p65 nuclear translocation, with the strongest effect observed after 30 min (Figure 4a). In line with these findings, LPS-induced expression of COX-2 was also downregulated by the treatment, reaching statistical significance at the 1:500 dilution (Figure 4b). Furthermore, analysis of pro-inflammatory cytokines in the conditioned media revealed a significant reduction in LPS-induced IL-1β and TNF-α release, starting at 1:1000 and 1:500 dilutions, respectively (Figure 4c,d).

### 3.4. Effect of the Dietary Supplement on Pyroptosis

**Figure 5:**
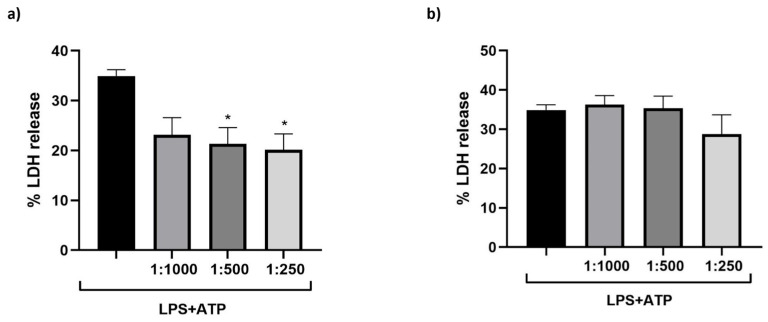
Effect of the dietary supplement on pyroptosis in cells treated with LPS + ATP. THP-1 cells were differentiated into M_0_ macrophage-like cells and subjected to sequential treatments designed to assess the effect of the dietary supplement on LPS + ATP-induced pyroptosis. (**a**) M_0_ macrophage-like cells were pre-treated with serial dilutions of the dietary supplement (1:1000, 1:500, 1:250) for 1 h. Cells were then stimulated with LPS (10 µg/mL) for 4 h, followed by ATP (5 mM) for 90 min. (**b**) M_0_ macrophage-like cells were first treated with LPS (10 µg/mL) for 4 h, subsequently incubated with the dietary supplement (1:1000, 1:500, 1:250) for 1 h, and finally stimulated with ATP (5 mM) for 90 min. Data are presented as mean ± SEM of three independent experiments performed in triplicate. * *p* < 0.05 vs. LPS + ATP.

To assess the effect of the dietary supplement on NLRP-3 inflammasome-mediated pyroptosis, M_0_ macrophages were stimulated with LPS (10 µg/mL for 4 h) and ATP (5 mM for 90 min). The supplement was administered either 1 h prior to LPS priming (Figure 5a) or 1 h prior to ATP stimulation (Figure 5b), at serial dilutions of 1:1000, 1:500, and 1:250. As shown in Figure 5a, treatment administered before LPS exposure significantly attenuated pyroptotic cell death, starting at the 1:500 dilution, compared to cells treated with the LPS + ATP (positive control). Conversely, administration prior to ATP stimulation resulted in a non-significant downward trend (panel b).

### 3.5. Effect of the Dietary Supplement on Granulocyte Cell Adhesion to HUVEC

One of the initial stages of inflammation is the stimulation of pro-inflammatory chemokines that induces diapedesis of granulocytes, whose adhesion to the vascular endothelium occurs first through mild selectin-mediated interactions and later through integrin-mediated strong adhesion. For these reasons, it was evaluated whether the supplement was able to modulate the adhesion of PMNs to the vessel endothelium. In order to assess the potential toxic effect of the supplement on primary endothelial cells (HUVEC), MTT assay was repeated under the same conditions of the previous experiments on THP-1 but increasing the time of the incubation of the cells until 72 h and as shown in Figure S1, the dietary supplement did not exert toxic effects.

**Figure 6:**
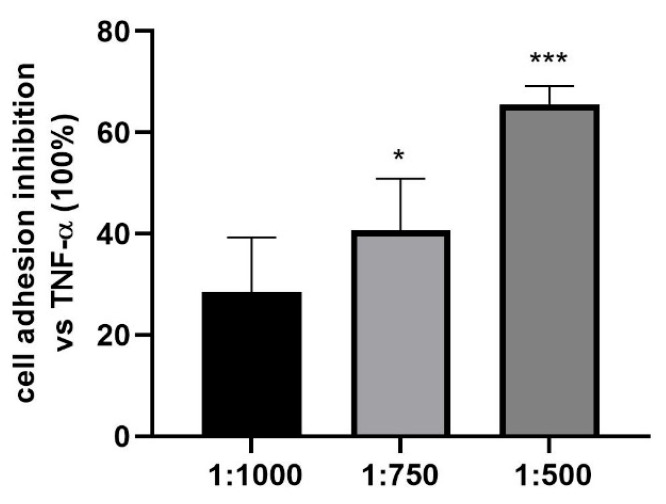
Effect of the dietary supplement on PMN adhesion to HUVEC treated with TNF-α. Human umbilical vein endothelial cells (HUVEC) were treated with serial dilutions of the dietary supplement (1:1000, 1:500, 1:250 for 48 h) and subsequently stimulated with TNF-α (10 nM, for 24 h). Polymorphonuclear cells (PMN) adhesion was then evaluated, and the results were expressed as percentage inhibition relative to the positive control (HUVEC pre-treated with TNF-α), which was set at 100% of adhesion. Data are presented as mean ± SEM from three independent experiments performed in triplicate. * *p* < 0.05, *** *p* < 0.001 vs. TNF-α-treated HUVECs (100% of adhesion).

To evaluate the effects of the treatment on PMN adhesion to HUVEC, HUVEC were treated for 48 h with different doses of the dietary supplement and stimulated with TNF-α (10 nM, 24 h), in order to activate the expression of adhesion molecules, and then the adhesion of PMNs. PMN adhesion inhibition was quantified relative to the positive control (TNF-α-treated HUVECs, set at 100% of adhesion). As shown in Figure 6, the supplement was able to significantly inhibit PMN adhesion to HUVEC starting from the 1:750 dilution.

### 3.6. Effect of the Dietary Supplement on Oxidative Stress

**Figure 7:**
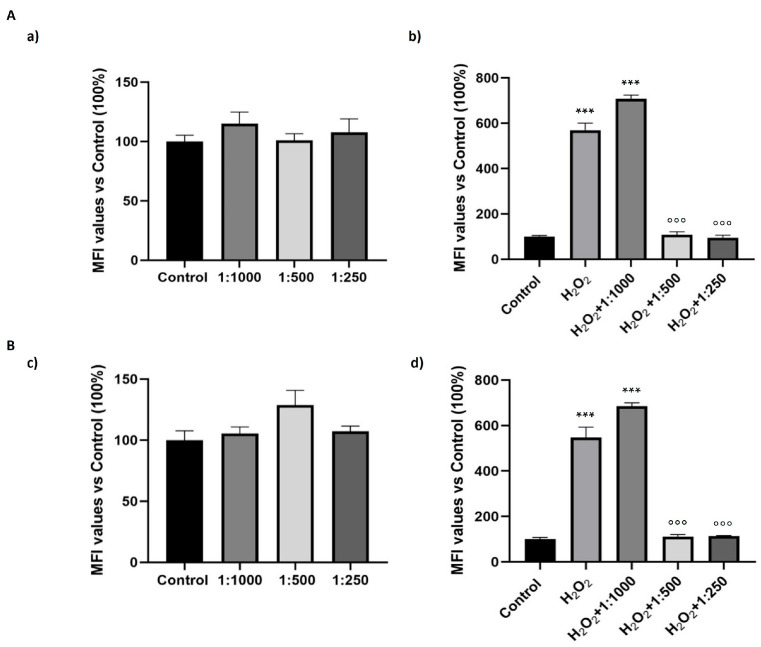
Effect of the dietary supplement on oxidative stress in human and mouse fibroblasts. Intracellular oxidative stress level was evaluated via DCFH-DA assay in human dermal fibroblasts (HDF, (**A**)) and L929 murine fibroblasts (**B**) after 24 h of treatments. (**a**,**c**) cells treated with the dietary supplement alone (dilutions: 1:1000, 1:500, 1:250). (**b**,**d**) cells treated with H_2_O_2_ alone (50 µM for HDF, 300 µM for L929) or in combination with the dietary supplement (dilutions: 1:1000, 1:500, 1:250). Bar graphs report Median Fluorescence Intensity (MFI) values as percentages relative to untreated control cells (Control, set at 100%). Data are shown as means ± SEM. *** *p* < 0.001 vs. Control; °°° *p* < 0.001 vs. H_2_O_2_ alone.

Fibroblasts are pivotal in sustaining tissue architecture and integrity. Dermal fibroblasts, in particular, are highly susceptible to oxidative stress, and their dynamic interplay with macrophages is fundamental to the regulation of tissue homeostasis. Indeed, their features are different but complementary. On this basis, we evaluated the antioxidant potential effect of the dietary supplement in human dermal fibroblasts and in mouse fibroblasts at dilutions that did not affect cell viability. As shown in Figure 7a,c, treatment with the dietary supplement alone at three different dilutions (1:1000, 1:500, and 1:250) did not increase intracellular oxidative stress levels in both cell lines. Interestingly, the supplement at the dilutions of 1:500 and 1:250 significantly exerted antioxidant effects in both cell lines treated with H_2_O_2_ (positive control), while at the highest dilution (1:1000) oxidative stress levels were similar to those observed in the positive control samples (Figure 7b,d).

### 3.7. Evaluation of the Presence of Dietary Supplement Ingredients in Cell Extracts

The presence of the ingredients of the formulation in cell extracts was evaluated using two different analytical platforms (HPLC-PDA-MS/MS and GC-MS) depending on the different components analyzed. Figure S2 shows representative chromatographic profiles of selected components detected in the dietary supplement. Of these, only some were found in the cell extracts treated with the dietary supplement, compared to the untreated cells: spermidine, 5-hydroxytryptophan (from *G. simplicifolia*), vitamins B_2_, B_5_, and vitamin E acetate, resveratrol, CoQ10, and bacosides (from *B. monnieri*). Figure S3 shows the representative chromatographic profiles of some of the above-mentioned compounds detected in the cell extracts treated with the dietary supplement compared with the untreated cells.

## 4. Discussion

The market for food supplements is rapidly expanding, offering a huge range of products (more than 90,000 in the USA alone) to consumers [18]. The global market size was valued at USD 177.50 billion in 2023 and is projected to grow at a compound annual growth rate of 9.1% from 2024 to 2030 [19]. The dietary supplements market of the United States is the largest, followed by Europe and Japan. Regarding Europe, in 2023, Italy recorded the highest market share in the European dietary supplements sector, accounting for 18.28%. Based on this share and projected market trends, the Italian market is estimated to reach approximately USD 11.6 billion by 2030 [20]. These data support the importance of dietary supplements in the country’s economy and underlying the persistent growth trend. Different factors may explain this phenomenon: the increase in people’s awareness about personal wellness and the crucial role of prevention; the growing availability of products able to address a huge range of health needs and the growing geriatric population, who are major consumers of dietary supplements [21]. Last but not least, the relationship of trust with people that suggest and/or sell these products, such as doctors, pharmacists, and herbalists.

In this context, we evaluated the effects of a multi-ingredient dietary supplement in human cells involved in tissue homeostasis maintenance.

We focus our attention on human macrophages, which are vital cells that function as transducers by translating information from the surrounding tissues in prompt reactions. Human macrophages constitute a highly dynamic cell type of the innate immune system, able to exert different functions like protection against pathogens, inflammation resolution, and tissue homeostasis maintenance [22].

Their plasticity allows macrophages to polarize to different phenotypes and then exert different functions in relation to a combination of factors including environmental cues, developmental origin, and duration spent in each tissue [6,23]. Thus, in function of the microenvironment they can be phenotypically polarized in two main groups: the classically activated macrophages or M_1_, which have a pro-inflammatory role and whose prototypical activating stimuli are IFNγ and LPS, and the alternatively activated macrophages or M_2_. The latter can be further subdivided into M_2a_, M_2b_ and M_2c_; we focused our attention on M_2a_ (after exposure to IL-4 or IL-13 as activating stimuli), the classically anti-inflammatory phenotype, that plays a crucial role in inflammation’s resolution [24,25].

Our results showed that the dietary supplement evaluated was able to modulate macrophage behavior. We observed an interesting dual anti-inflammatory effect: the supplement not only may reduce the invasion of the inflamed tissue by the inflammatory phenotype M_1_, but it is also able to increase the migration of M_2_ macrophages. A similar result has already been achieved in our laboratory [10] and highlights the known different migration properties of M_1_ and M_2_ macrophages [26,27], but is very surprising for a dietary supplement. In addition, as shown in Figure 3, we also demonstrated that the treatment promoted the activation of M_0_ cells to M_2_ phenotype, the anti-inflammatory one, thus explaining the reason for an increased cell migration induced by the dietary supplement in M_0._ Therefore, the dietary supplement increased the enrolment of M_2_ cells, promoting both their activation and their migration.

To test whether the supplement could have a beneficial effect also in inflammatory conditions, we investigated the effect of the dietary supplement on macrophages treated with LPS as a pro-inflammatory stimulus. Interestingly, the treatment was able to reduce the expression of COX-2, the inducible form of cyclooxygenases overexpressed in inflammatory conditions and, accordingly, reduced the level of the pro-inflammatory cytokines IL-1β and TNF-α. The mechanism underlying the observed effects involves the suppression of NF-κB activation, a key transcription factor responsible for the expression of various inflammatory mediators, such as COX-2, TNF-α, and IL-1β [28]. NF-κB is a central mediator of pro-inflammatory gene induction and functions in both innate and adaptive immune cells [29,30]. Its canonical activation depends on inducible degradation of IκBα, which normally prevents NF-κB dimers from binding DNA and maintains their cytoplasmic localization. Upon IκBα degradation, NF-κB complexes, primarily p50/p65, translocate rapidly to the nucleus [31]. Our results showed that p65 nuclear translocation was induced by LPS treatment but interestingly counteracted by the supplement with a significant effect after 30 min.

NF-κB contributes to the initiation and development of inflammatory diseases and it is also involved in the regulation of NLRP-3 inflammasome activation, the most extensively studied inflammasome [32]. Notably, NF-κB is a central mediator of the priming signal for its activation [33] inducing the transcriptional expression of NLRP-3 and pro-IL-1β. The priming signal (signal 1) is followed by an activation signal (signal 2) required to promote the release of IL-1β and to induce pyroptosis mediated by NLRP-3 [34].

Our results showed that treatment with the dietary supplement could significantly reduce NLRP-3 inflammasome-induced pyroptotic cell death starting at the dilution of 1:500 when the dietary supplement was added before LPS but was ineffective when it was added before the second stimulus, ATP. This data suggested, as quite expected, that the dietary supplement is not a specific NLRP-3 inflammasome inhibitor but was able to counteract the pyroptosis NLRP-3 mediated when added before LPS, likely inhibiting the priming effect induced by NF-κB. Consistently, we can speculate that the supplement acts at an early regulatory step acting upstream in the NLRP-3 activation cascade. These data also confirmed the ability of the dietary supplement to inhibit p65 nuclear translocation, providing additional insight into its role in preserving tissue homeostasis and counteracting the development of low-grade chronic inflammation.

Interestingly, these effects could be ascribed to different ingredients of the formulation. Recent studies supported anti-inflammatory properties for CoQ10 [35,36]. In particular, Bessler et al. [37] demonstrated a significant reduction in TNF-α and IL-2 release in human peripheral blood mononuclear cells (PBMC) treated for 24 h at micromolar concentrations, that are fully comparable with the concentration of CoQ10 obtained in our model. Interestingly, the anti-inflammatory properties of CoQ10 were also confirmed in clinical trials. An interesting metanalysis including 17 RCT trials showed that CoQ10 supplementation significantly reduced the levels of circulating C-reactive protein (CRP), interleukin-6 (IL)-6 and TNF-α [38]. The effect on TNF-α was also evident from another metanalysis that evaluated the anti-inflammatory effect of CoQ10 on people affected by metabolic diseases [39]. The mechanisms underlying these effects are not completely understood but the involvement of the inhibition of NF-κB signaling pathways was demonstrated [40,41]. The ability to counteract this pro-inflammatory transcriptional factor has also been demonstrated for spermidine, a polyamine, naturally present in our body. Compelling evidence has indicated that spermidine could affect macrophage behavior, especially by promoting the M_2_ phenotype [42,43]. Again, these effects were observed at micromolar concentrations of spermidine, comparable to the concentration used in our experiment. Interestingly, a relevant paper demonstrated that oral supplementation of spermidine can extend the lifespan in rodents and indicated that high levels of dietary spermidine are associated with a lower incidence of cardiovascular diseases in humans [44]. In particular, the authors underlined that high levels of spermidine were associated with lower circulating levels of TNF-α and IL-1β. Moreover, Guo et al. [45] showed for the first time that spermidine was able to inhibit cell pyroptosis. The authors, in this paper evaluating the effects of spermidine in an in vivo model of osteoarthritis demonstrated that treatment was able to inhibit inflammation and pyroptosis by modulating the NF-κB and NLRP-3 cascades.

An active role could also be played by the extract of *Bacopa monnieri,* a plant used in ayurvedic medicine in India for more than 3000 years to treat various inflammation-related diseases. Evidence on anti-inflammatory effects for Bacopa is derived from both in vitro [46] and in vivo studies, especially in rodent experimental models of inflammation [47,48,49] or neuroinflammation [50,51] as well as from clinical trials [52]. Regarding vitamins, the anti-inflammatory properties of vitamins B_2_ and B_5_ are already known. In particular, in vitro studies demonstrated that vitamin B_2_ is able to modulate granulocyte migration [53] and its deprivation has negative effects on both the activity and viability of macrophages [54], whereas in vivo studies showed that IV infusion of vitamin B_2_ in mice decreased the levels of pro-inflammatory cytokines and nitric oxide induced by lipopolysaccharide [55]. Finally, evidence supporting an anti-inflammatory effect in ex vivo studies on PBMC [56] or in vitro dendritic cells [57], as well as in patients [58] are also published for vitamin E supplementation. Beyond monocytes/macrophages, another kind of cells critical for the initiation, maintenance, and resolution of inflammation are neutrophils [59,60]; thus, we have also examined whether the dietary supplement could modulate neutrophil adhesion to endothelial cells. Neutrophils are the first immune cells to arrive in the damaged tissues via the vasculature [61]. The first step of the adhesion cascade consists of an initial attachment of the neutrophil to the activated endothelium and finishes with a transmigration of the neutrophil into the tissue, where neutrophils may exert their functions [59,62]. We observed the effect of dietary supplement pretreatment on the primary step in leukocyte recruitment: the adhesive interactions between neutrophils and endothelial cells of inflamed tissue, and our data showed that after exposure to the pro-inflammatory stimulus (TNF-α), endothelial cells were less suitable for the adhesion of neutrophils if pretreated with the dietary supplement. In fact, we observed a significant reduction at the dose of 1:750 and a maximum effect at the higher dose, which inhibited cell adhesion by up to 80%. This effect could have important significance because it highlights the potential utility of a supplementation of bioactive ingredients in the reduction in the inflammatory response, for both the inhibition of granulocytes adhesion and more generally for the reduction in the reactivity of the activated endothelium. Reducing endothelial reactivity helps to maintain the vascular homeostasis [63].

After migration to extravascular space, leukocytes interact with other types of cells, including fibroblasts. These cellular communications may contribute not only to tissue regeneration processes but also to the amplification of chronic inflammatory responses. Human dermal fibroblasts help in maintaining tissue homeostasis and improving wound repair through the synthesis of extracellular matrix proteins [64,65]. Reactive oxygen species (ROS) are indispensable to protect the organism from invading microorganisms. However, immoderate production of ROS is recognized as a causative factor for aging and several other pathological conditions. In this study, after excluding any pro-oxidant effect, we demonstrated that treatment with the dietary supplement was able to protect dermal fibroblasts against ROS accumulation induced by H_2_O_2_. We observed this effect on both human and mouse fibroblasts. Again, different ingredients could have a significant role in this effect. In particular, several studies have demonstrated the beneficial role of resveratrol in oxidative stress-induced skin cellular senescence [66], thus supporting that it could play a role in the antioxidant effects mediated by the supplement in fibroblasts. Beyond the reduction in ROS and the increase in the activity of antioxidant enzymes, it was recognized that resveratrol can modulate the NF-κB pathway, the activation of MAPK cascades and the expression of COX-2, as well as the release of cytokines [67,68]. Its antioxidant and anti-inflammatory activity have been demonstrated at all levels starting from in vitro to human studies [68]. In addition, an active role could also be exerted by the seeds of *Griffonia simplicifolia* (a tropical medicinal plant native to West and Central Africa) that are the main natural source of 5-HTP, a known precursor of serotonin [69]. Several studies found that 5-HTP is a potent mediator against inflammation. For instance, it was demonstrated to reduce the activation of MAPKs and NF-κB signals in fibroblasts and reduce the release of pro-inflammatory cytokines in both in vitro and in vivo models [70,71,72].

Our study identified in cell extracts only some of the active ingredients of the formulation, these could be due to both detection threshold and metabolization. Therefore, we cannot rule out a causative role in the observed effects, also for the ingredients that we were unable to identify in cell extracts. Indeed, one of the main points of strength of this product is its multi-ingredient composition. Having analyzed the effect of the dietary supplement in its totality, we are unable to identify the effects derived from each component, but we can speculate that they could work in synergy. For example, niacin together with ribose, vitamin B_12_, vitamin B_1_, and with *Griffonia* (that in providing tryptophan, which is an essential precursor of NAD) can play a crucial role in cell metabolism. Cellular homeostasis also regulates the NAD/NADH ratio and the latter, in turn, is fundamental for the reduction of CoQ10 to its active form, ubiquinol, which is crucial for the synthesis of ATP.

This study presents a limitation regarding cellular models. We performed experiments by treating the cells with specific stimuli to achieve M_1_ or M_2_ macrophages, to represent the paradigm of the pro-inflammatory and the anti-inflammatory phenotypes, respectively. However, the in vivo system complexity is huge: the signals encountered by cells are diverse, both temporally and spatially, and macrophages not only respond with equally diverse phenotypes but also can switch from a ‘functional’ phenotype to another. Unfortunately, their plasticity, a characteristic feature of these cells, is very difficult to reproduce in in vitro systems.

Moreover, although cellular models provide valuable tools to dissect and understand specific biological mechanisms, they inevitably cannot completely reproduce the full complexity of human physiology. Importantly, in vitro models are inherently unsuitable for assessing pharmacokinetic parameters such as bioavailability, metabolism, and systemic distribution of the compounds under investigation.

Within this context, another limitation concerns the concentration used, as this compound has never been investigated in animal studies or clinical trials. Consequently, there is no available data on its plasma concentrations following oral administration, making it impossible to directly compare the doses used in vitro with those that would be physiologically achievable in animals or humans. Therefore, the next step will be to test the dietary supplement in animal models to validate its biological effects and assess its pharmacokinetics in vivo. These studies will help inform the design of future clinical trials, which will be essential to confirm the clinical relevance and translational applicability of our findings.

In this study, antioxidant assessment was conducted using the DCF-DA assay, a well-established and sensitive technique for monitoring intracellular ROS fluctuations. While this method is widely employed, we recognize that a more comprehensive evaluation including enzymatic markers such as superoxide dismutase, and glutathione peroxidase would offer deeper mechanistic insights and bolster the robustness of our conclusions. Nevertheless, the antioxidant properties of key bioactive components integrated in the formulation, including CoQ10 and resveratrol, are well-supported by the existing literature.

This study was specifically designed to assess the biological activity of the complete multi-ingredient dietary supplement, without aiming to compare its formulation or isolate the effects of its individual constituents. Consequently, potential synergistic interactions among its components remain unexplored and were outside the scope of this work. Further research is needed to unravel these interactions, elucidate the individual contributions of each compound, and benchmark this formulation against other mono- or multi-ingredient supplements.

In contrast, our strong point is to have evaluated the effect of a dietary supplement on human cells, a very important step to understand the mechanism underlying the beneficial effect perceived by consumers and that unfortunately, is too rarely investigated.

## 5. Conclusions

In conclusion, it is well known that the degradation of tissue homeostasis or the impossibility of restoring it after an injury can have deleterious effects, inducing uncontrolled chronic inflammation and increased oxidative stress. Indeed, reducing inflammation is recognized as one of the ways to reduce the risk of many age-related diseases such as cardiovascular disease, diabetes, rheumatoid arthritis, and cancer.

Thus, our findings demonstrate how a combination of natural active ingredients may support the maintenance of balance within the cellular inflammatory-homeostatic signaling axis.

Pivotal results were presented at the XIV National Congress of SINut (Bologna, Italy, 12–14 September 2024; abstract published in Pharmanutrition and Functional Food) and at the 42nd National Congress of SIF (Sorrento, Italy, 13–16 November 2024).

## Figures and Tables

**Table 1:** Oligonucleotide sequence of primers used for qRT-PCR.

Primer	Sense	Reverse
Human CD80	5′-CCTACTGCTTTGCCCCAAGA-3′	5′-CAGGGCGTACACTTTCCCTT-3′
Human CD86	5′-TGGAAACTGACAAGACGCGG-3′	5′-CAAGGAATGTGGTCTGGGGG-3′
Human MRC1	5′-CAGCGCTTGTGATCTTCATT-3′	5′-TACCCCTGCTCCTGGTTTTT-3′
Human CD163	5′-GCAGTTTCCTCAAGAGGAGAGAA-3′	5′-GCTCAGAATGGCCTCCTTTTC-3′
Human GAPDH	5′-TGGTATCGTGGAAGGACTCATGAC-3′	5′-ATGCCAGTGAGCTTCCCGTTCAGC-3′

**Table 2:** Sample preparation and analytical details for the analysis of the dietary supplement components. * SRM: selected reaction monitoring; SIM: single ion monitoring.

Sample Preparation	Analytical Platform	Stationary Phase	Gradient Program	Detection Mode *	Component
Extraction in MeOH 20%	HPLC-MS	Ascentis Express C18 column (15 cm × 2.1 mm, 2.7 μm, Supelco, Bellefonte, PA, USA)	5% B for 3 min, 5–15% B in 17 min, 15–25% B in 10 min, 25–70% B in 12 min, 70–100% B in 10 min, 100% B for 1 min	SRM^+^ (265→122)	Vitamin B1
				SRM^+^ (123→80)	Vitamin B3
				SRM^+^ (146→72)	Spermidine
				SRM^+^ (221→134)	5-hydroxytryptophan
				SRM^+^ (220→70)	Vitamin B5
				SRM^+^ (337→172)	Vitamin B2
Extraction in MeOH 100%	HPLC-MS	Ascentis Express C18 column (15 cm × 2.1 mm, 2.7 μm, Supelco, Bellefonte, PA, USA)	5% B for 3 min, 5–15% B in 17 min, 15–25% B in 10 min, 25–70% B in 12 min, 70–100% B in 10 min, 100% B for 1 min	SIM^+^ (943, 973)	Bacosides
Dilution in EtOH 100%	HPLC-PDA	Ascentis Express RP-Amide column (10 cm × 2.1 mm × 2.7 μm, Supelco, Bellefonte, PA, USA)	25% B for 3 min, 25–70% B in 12 min, 70–100% B in 10 min, 100% for 25 min	UV (λ = 305 nm)	Resveratrol
				UV (λ =274 nm)	Coenzyme Q10

## Data Availability

The original contributions presented in this study are included in the article/Supplementary Materials. Further inquiries can be directed to the corresponding author.

## Supplementary Materials

The following supporting information can be downloaded at: https://www.mdpi.com/article/10.3390/nu17162587/s1, Table S1: Ingredients of the dietary supplement used in the study; Figure S1: Effect of the dietary supplement on cell growth in HUVEC. Figure S2: Representative chromatographic profiles of the selected compounds contained in the dietary supplement; Figure S3: Representative chromatographic profiles of some compounds detected in the cell extracts treated with the dietary supplement compared with the untreated cells.
