# Supplementary material for: Balancing the Cellular Inflammatory-Homeostatic Axis Through Natural Ingredient Supplementation
Source: Nutrients. 2025 Aug 8;17(16):2587. doi: 10.3390/nu17162587 (PMC12389121; doi:10.3390/nu17162587)
Supplement: Supplementary file 1 [file nutrients-17-02587-s001.zip › nutrients-3752571-supplementary.pdf]

SUPPLEMENT FACTS		
**INGREDIENTS**	**DAILY DOSE**	**% DAILY VALUE**
Coenzyme Q10	200 mg	**
D-Ribose	150 mg	**
Phosphotidylserine 50%	100 mg	**
Resveratrol	100 mg	**
Vitamin B3 (niacin)	54 mg	337,5
Griffonia simplicifolia (seed extract)	50 mg	**
Bacopa monnieri (leaves extract)	30 mg	**
Vitamin C (L-ascorbic acid)	30 mg	37,5
Zinc Gluconate	15 mg	150
Vitamin E (tocopheryl acetate)	12 mg	100
Vitamin B6 (pyridoxine hydrochloride)	10 mg	714,2
Spermidine	6 mg	**
Vitamin B5 (pantothenic acid)	6 mg	100
Sodium	5 mg	<1
Vitamin B2 (riboflavin)	1,4 mg	100
Vitamin B1 (thiamine)	1,1 mg	100
Vitamin B12 (cyanocobalamin)	1 mg	40.000
Vitamin B9 (folate)	400 mcg	200
Selenium (selenomethionine)	55 mcg	100
** Percent Daily Value are based on a 2000-calorie diet<br>** Daily Value Not Established		

Other ingredients: dibasic calcium phosphate (as bulking agent); magnesium salt of fatty acids (as anticaking agent).

Table S1. Ingredients of the dietary supplement under investigation in this study.
The table reports the qualitative and quantitative composition of the dietary supplement. The relative amount for each ingredient is referred to the recommended daily dose (2 capsules).

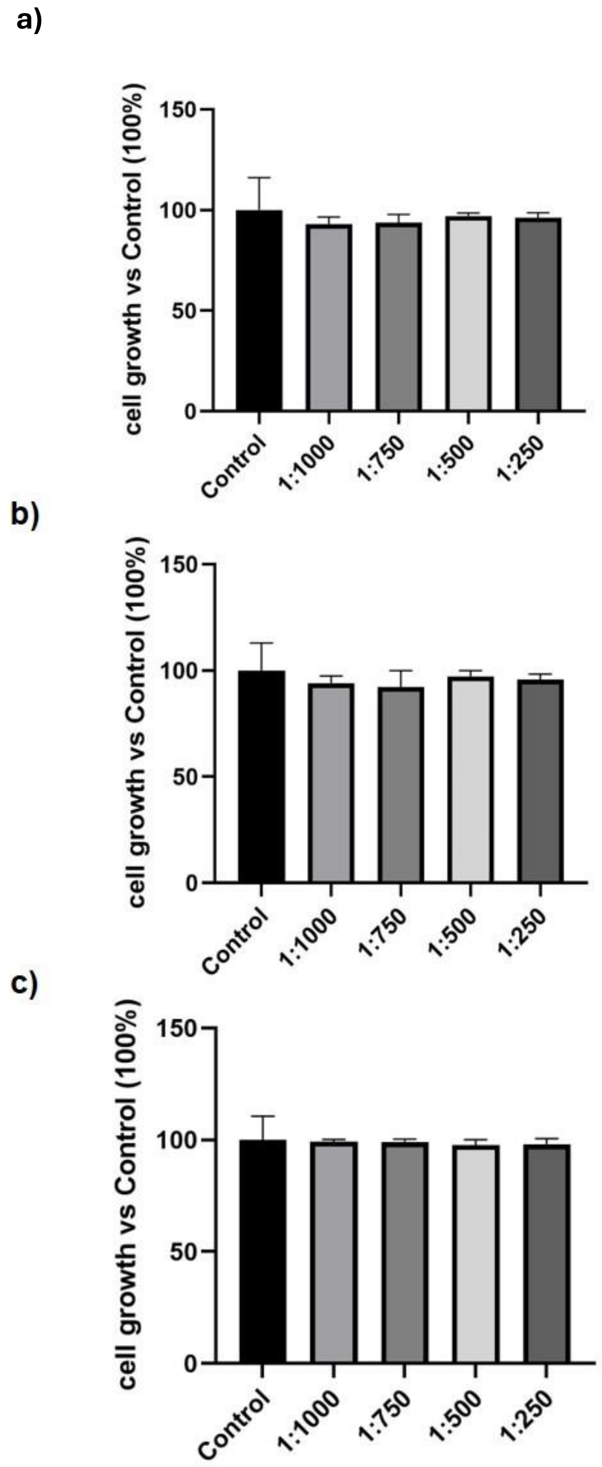

Figure S1. Effect of the dietary supplement on cell growth in HUVEC.

HUVEC were exposed to either vehicle alone (Control) or serial dilutions of the supplement. Cell growth was measured by MTT assay at 24 h (panel a), 48 h (panel b) or 72 h (panel c) and was expressed as percentage vs control (100%). Data are expressed as mean ± SEM of three independent experiments conducted in triplicate.

A

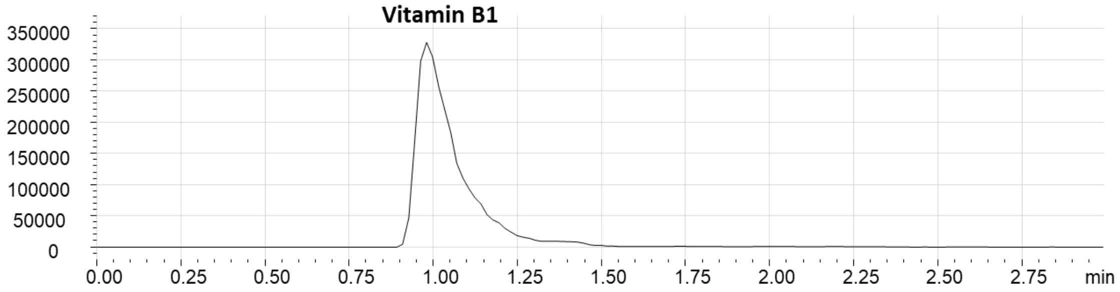

Vitamin B1
350000
300000
250000
200000
150000
100000
50000
0
0.00 0.25 0.50 0.75 1.00 1.25 1.50 1.75 2.00 2.25 2.50 2.75 min

B

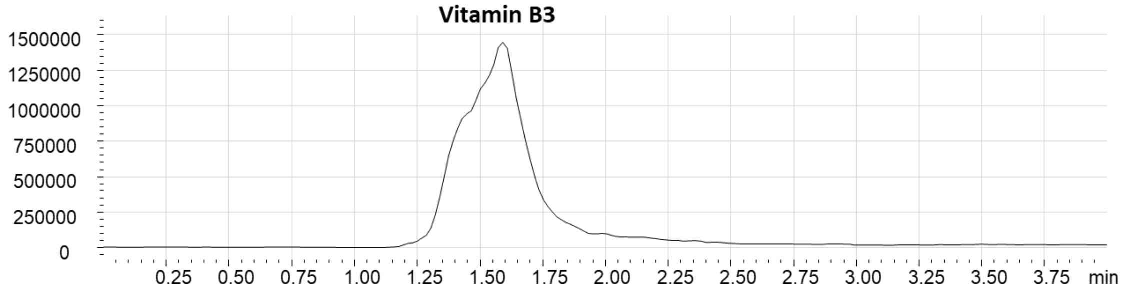

Vitamin B3
1500000
1250000
1000000
750000
500000
250000
0
0.25 0.50 0.75 1.00 1.25 1.50 1.75 2.00 2.25 2.50 2.75 3.00 3.25 3.50 3.75 min

C

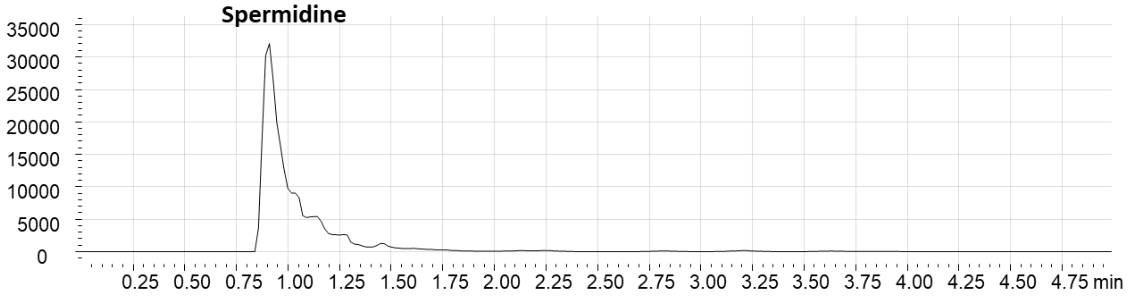

Spermidine
35000
30000
25000
20000
15000
10000
5000
0
0.25 0.50 0.75 1.00 1.25 1.50 1.75 2.00 2.25 2.50 2.75 3.00 3.25 3.50 3.75 4.00 4.25 4.50 4.75 min

D

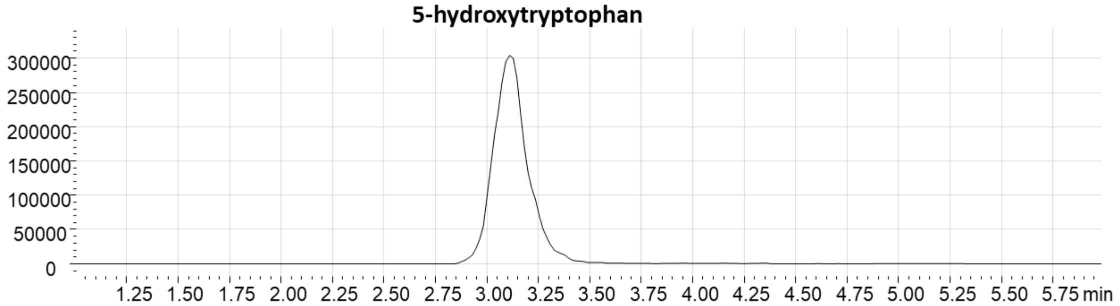

5-hydroxytryptophan
300000
250000
200000
150000
100000
50000
0
1.25 1.50 1.75 2.00 2.25 2.50 2.75 3.00 3.25 3.50 3.75 4.00 4.25 4.50 4.75 5.00 5.25 5.50 5.75 min

E

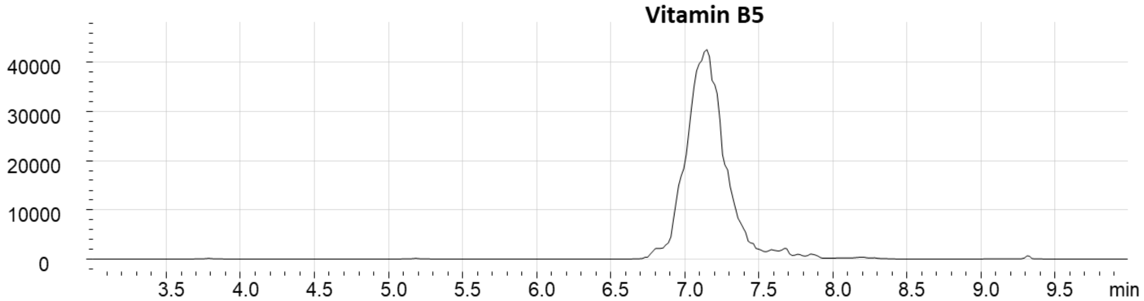

Vitamin B5
40000
30000
20000
10000
0
3.5 4.0 4.5 5.0 5.5 6.0 6.5 7.0 7.5 8.0 8.5 9.0 9.5 min

F

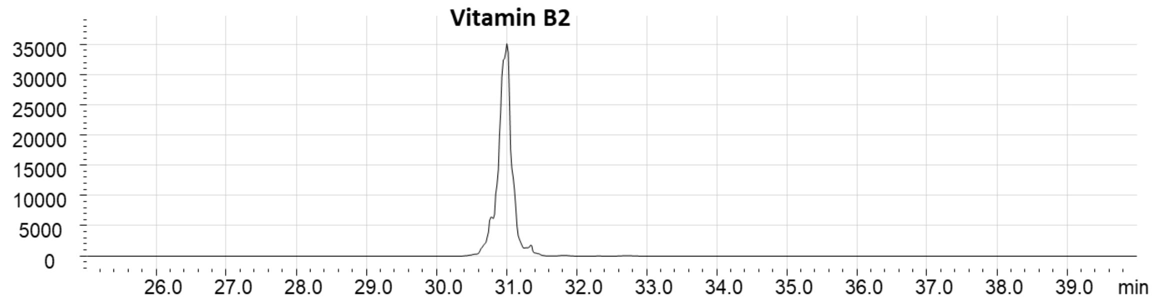

G

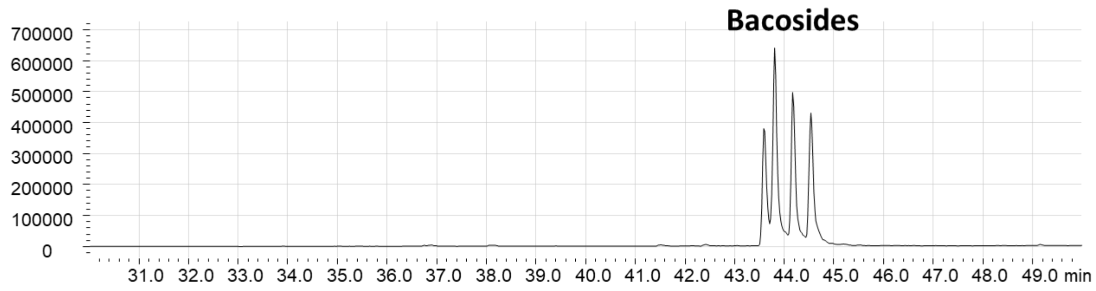

H

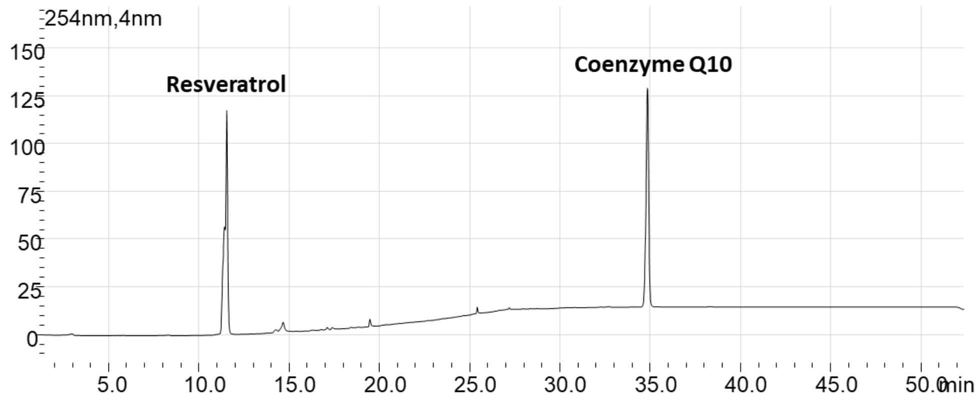

I

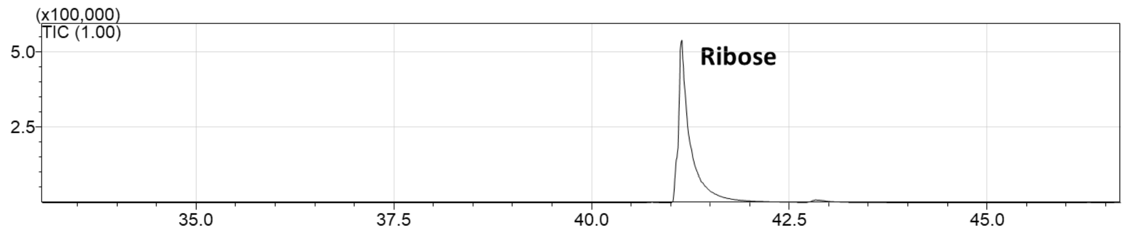

L

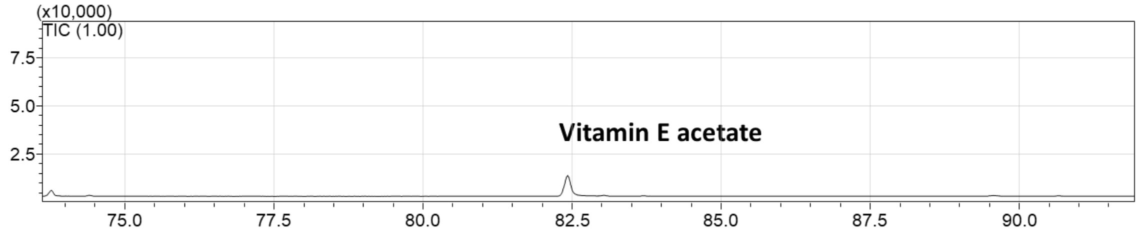

Figure S2. Representative chromatographic profiles of the selected compounds contained in the dietary supplement. Their identification was confirmed with the co-injection of the corresponding commercial standards (A) Vitamin B1 analyzed in HPLC-MS, RT 0.981 min, SRM mode (265→122); (B) Vitamin B3

analyzed in HPLC-MS, SRM mode (123→80), RT 1.574 min; (C) spermidine analyzed in HPLC-MS, SRM mode (146→72), RT 0.910 min; (D) 5-hydroxytryptophan analyzed in HPLC-MS, SRM mode (221→134), RT 3.111 min; (E) Vitamin B5 analyzed in HPLC-MS, SRM mode (220→70), RT 7.134 min; (F) Vitamin B2 analyzed in HPLC-MS, SRM mode (337→172), RT 30.987 min; (G) bacosides analyzed in HPLC-MS, SIM mode (943, 973), RT 43.613 min, 43.835 min, 44.200 min, 44.584 min; (H) resveratrol and coenzyme Q10 analyzed in HPLC-PDA, resveratrol: λmax: 305/317 nm, RT 11.601 min, coenzyme Q10: λmax: 274 nm, RT 34.871 min; (I) ribose analyzed in GC-MS, SIM mode (307), RT 41.127 min; (L) vitamin E acetate analyzed in GC-MS, SIM mode (165), RT 82.447 min. More details of the method of analysis can be found in Table X.

A

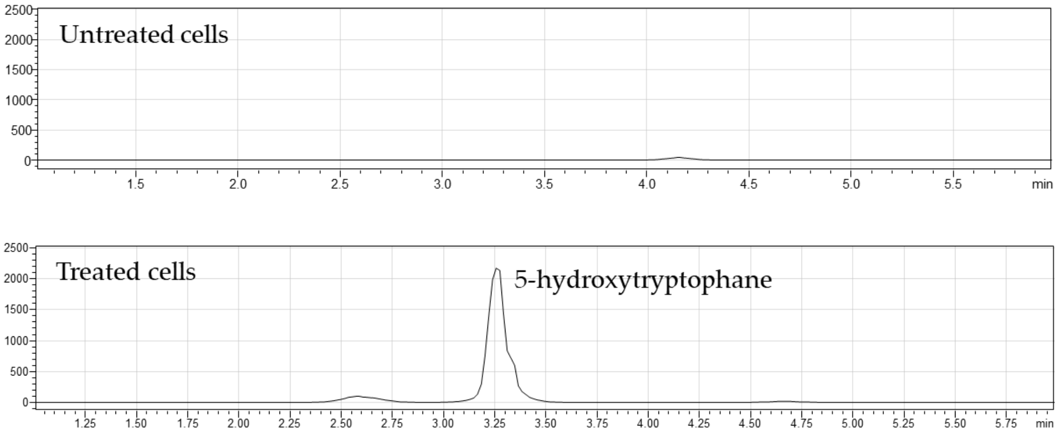

B

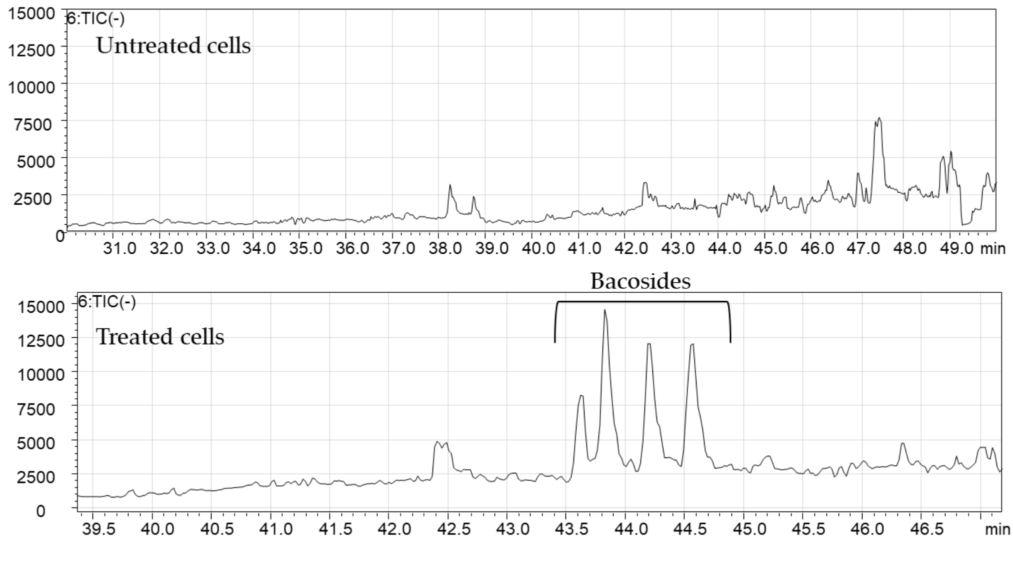

C

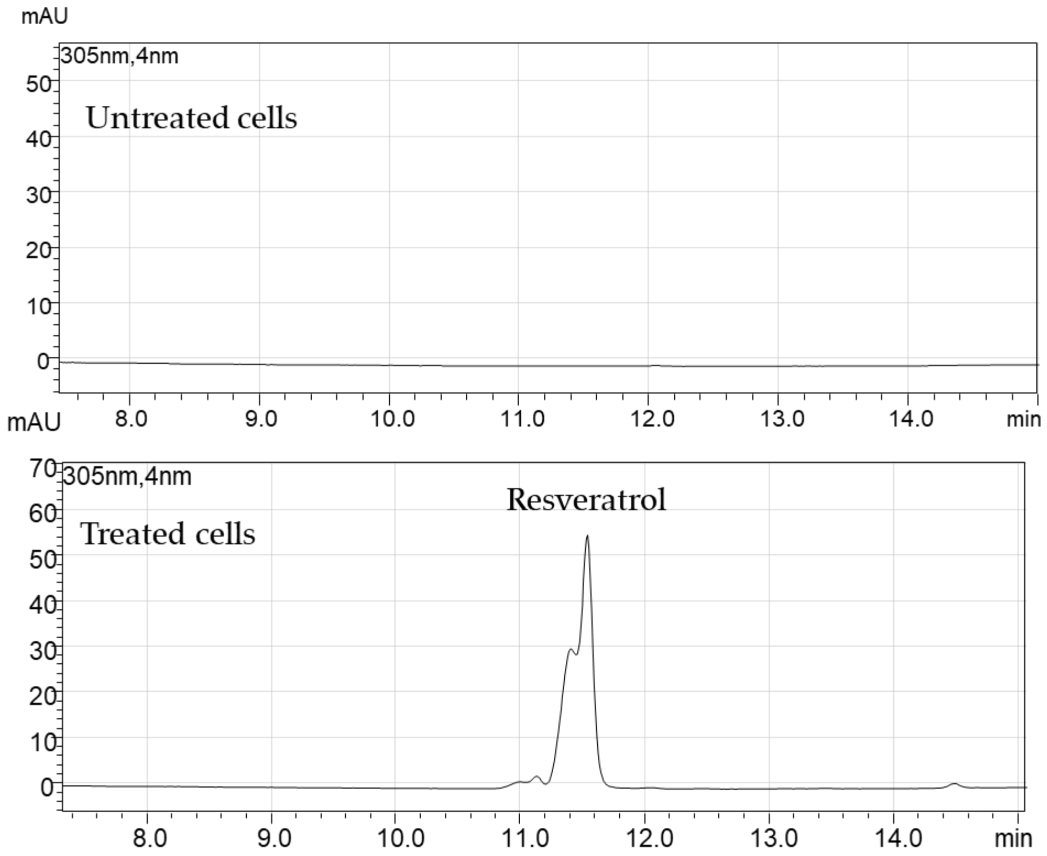

D

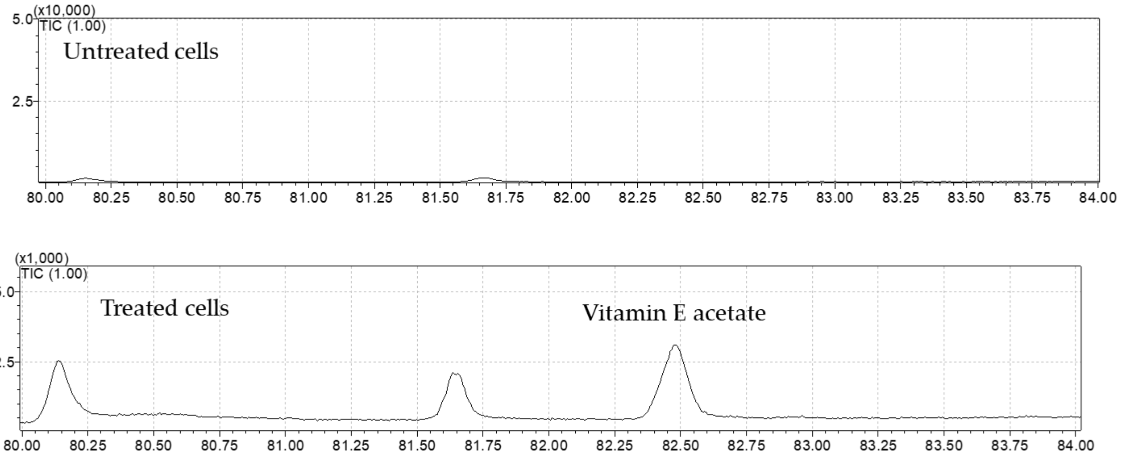

Figure S3. Representative chromatographic profiles of some compounds detected in cell extracts treated with the dietary supplement compared with the untreated cells. (A) 5-hydroxytryptophane; (B) Bacosides; (C) resveratrol; (D) vitamin E acetate.
